# Prenatal Endotoxemia and Placental Drug Transport in The Mouse: Placental Size-Specific Effects

**DOI:** 10.1371/journal.pone.0065728

**Published:** 2013-06-10

**Authors:** Enrrico Bloise, Manzerul Bhuiyan, Melanie C. Audette, Sophie Petropoulos, Mohsen Javam, William Gibb, Stephen G. Matthews

**Affiliations:** 1 Department of Physiology, University of Toronto, Toronto, Ontario, Canada; 2 Department of Pharmacology and Therapeutics, McGill University, Montreal, Quebec, Canada; 3 Department of Obstetrics and Gynecology, University of Ottawa, Ottawa, Ontario, Canada; 4 Department of Cellular and Molecular Medicine, University of Ottawa, Ottawa, Ontario, Canada; 5 Department of Obstetrics and Gynecology, University of Toronto, Toronto, Ontario, Canada; 6 Department of Medicine, Faculty of Medicine, University of Toronto, Toronto, Ontario, Canada; Institut Jacques Monod - UMR 7592 CNRS - Université Paris Diderot, France

## Abstract

Lipopolysaccharide (LPS) in high doses inhibits placental multidrug resistance P-glycoprotein (P-gp - *Abcb1a/b*) and breast cancer resistance protein (BCRP - *Abcg2*). This potentially impairs fetal protection against harmful factors in the maternal circulation. However, it is unknown whether LPS exposure, at doses that mimic sub-lethal clinical infection, alters placental multidrug resistance. We hypothesized that sub-lethal (fetal) LPS exposure reduces placental P-gp activity. **Acute** LPS (n = 19;150 µg/kg; ip) or vehicle (n = 19) were given to C57BL/6 mice at E15.5 and E17.5. Placentas and fetal-units were collected 4 and 24 h following injection. **Chronic** LPS (n = 6; 5 µg/kg/day; ip) or vehicle (n = 5) were administered from E11.5–15.5 and tissues were collected 4 h after final treatment. P-gp activity was assessed by [^3^H]digoxin accumulation. Placental *Abcb1a/b*, *Abcg2*, *interleukin-6* (*Il-6*), *Tnf-α, Il-10* and *toll-like receptor-4* (*Tlr-4*) mRNA were measured by qPCR. Maternal plasma IL-6 was determined. At E15.5, maternal IL-6 was elevated 4 h after single (*p*<0.001) and chronic (*p*<0.05) LPS, but levels had returned to baseline by 24 h. Placental *Il-6* mRNA was also increased after acute and chronic LPS treatments (*p*<0.05), whereas *Abcb1a/b* and *Abcg2* mRNA were unaffected. However, fetal [^3^H]digoxin accumulation was increased (*p*<0.05) 4 h after acute LPS, and maternal [^3^H]digoxin myocardial accumulation was increased (*p*<0.05) in mice exposed to chronic LPS treatments. There was a negative correlation between fetal [^3^H]digoxin accumulation and placental size (*p*<0.0001). Acute and chronic sub-lethal LPS exposure resulted in a robust inflammatory response in the maternal systemic circulation and placenta. Acute infection decreased placental P-gp activity in a time- and gestational age-dependent manner. Chronic LPS decreased P-gp activity in the maternal myocardium and there was a trend for fetuses with smaller placentas to accumulate more P-gp substrate than their larger counterparts. Collectively, we demonstrate that acute sub-lethal LPS exposure during pregnancy impairs fetal protection against potentially harmful xenobiotics in the maternal circulation.

## Introduction

Inflammatory processes can occur in response to a wide variety of pathological stimuli, including infection, tissue damage, trauma and cellular stress. These processes can elicit the release of a number of pro-inflammatory cytokines involved in immunological responses and tissue homeostasis [1]. During pregnancy, a dysregulation of cytokine networks can lead to sub-clinical and clinical chorioamnionitis [2], spontaneous abortion, preterm delivery (PTD), preeclampsia and intrauterine growth restriction [3]. Additionally, neonates born with inflammatory related conditions are at an increased risk for short- and long-term complications, including neonatal encephalopathy, long-term cognitive impairment and cerebral palsy [1], [4].

In this context, multidrug resistance transporters such as P-glycoprotein (P-gp; encoded by *Abcb1a/b* genes in the mouse) and breast cancer resistance protein (BCRP; encoded by *Abcg2* gene), are functionally modulated by pro-inflammatory agents in different tissues/cell types [5]–[10]. P-gp and BCRP are extensively expressed in different tissue barriers such as the placenta, blood-brain barrier, mammary gland, colon and small intestine [11]. They play an important physiological role in extruding a large number of cellular substrates, such as small organic cations, carbohydrates, polysaccharides and proteins [8]. In addition to extruding physiological substrates, multidrug resistance proteins also extrude a wide range of xenobiotics such as chemotherapeutics and environmental toxins [12].

P-gp and BCRP are expressed on the microvillous (maternal blood-facing) membrane of the syncytial trophoblast cell layer [13], [14], while BCRP is also expressed in fetal blood vessels of the villous core [15], [16]. P-gp and BCRP restrict the entry of factors present in the maternal circulation to the fetus [17]. Consequently, in cases where the mother is undergoing acute or chronic inflammatory related processes, altered P-gp and BCRP activity could potentially impair fetal protection against harmful factors in the maternal circulation. In particular, previous studies have demonstrated that acute exposure to very high doses of lipopolysaccharide (LPS - a major component of the outer membrane of gram-negative bacteria) inhibits placental P-gp activity and expression in the rat [6], [7]. However, it was unclear in these studies how high dose LPS treatments (given at E17 for 24 h; ranging from 100 to 1000 µg/Kg,) impacted pregnancy outcome and fetal survival. Indeed, lower doses of LPS (given at E14.5 for 72 h; ranging 50 to 100 µg/Kg) have been demonstrated to reduce fetal survival rates in the rat [18]. As such, it is not known whether maternal infection/inflammation, at a magnitude compatible to fetal survival, can decrease placental P-gp activity in late gestation and thus alter fetal drug exposure of neonates. Therefore, we conducted a series of experiments to determine whether sub-lethal maternal inflammation is capable of altering transplacental transfer of P-gp substrates in late gestation. We used a mouse model of induced maternal systemic endotoxemia that results in less than 20% of fetal death/reabsorption.

We also examined placental P-gp transport efficiency within a litter. There is a natural variation in placental size in polytocus animals that almost certainly mirrors variations in the fetal to placental (F:P) weight ratio [19]. The F:P ratio is an important indicator of placental efficiency, where a higher F:P weight ratio indicates that the placenta supports more fetal growth per unit of placenta. [19], [20]. For example, the activity and expression of active transporters, such as the Na^+^-dependent system A neutral amino acid transporter, is increased in the smallest placenta when compared to the largest placenta [21]. Furthermore, in cases where placental growth trajectory is altered, the efficiency of this transporter is also modified [22], demonstrating a strong correlation between placental size and placental transport efficiency. Here, we also evaluated if P-gp expression and activity are placental size-dependent and how chronic maternal systemic inflammatory stimuli impact placental P-gp activity, taking into account natural variation in the F:P weight ratio within a litter.

## Materials and Methods

### 2.1. Animals

Virgin female C57BL/6 mice (6–8 weeks – n = 65; Charles River, Germantown, New York) were bred (with C57BL/6 male mice – n = 12) as previously described [23]. After the presence of a vaginal plug (Embryonic day [E]0.5), pregnant mice were randomly assigned to treatment groups receiving either LPS or vehicle. These studies were conducted using protocols approved by the Animal Care Committee at the University of Toronto and in accordance with the Canadian Council for Animal Care. The Animal Care Committee at the University of Toronto has specifically approved this study.

### 2.2. LPS Dose Titration

Acute and chronic LPS dose titration experiments were performed in order to determine the LPS treatment doses that produced physiological systemic inflammatory responses, but did not produce more than 20% fetal death/reabsorption rates. For the acute experiments, pregnant mice received a single dose of LPS (L2880– Sigma, St Louis, MO) at the following concentrations: 20, 40, 50, 80, 100, 120, 150 or 200 µg/Kg; i.p.; on E14.5 and the ratio of dead/reabsorbed fetuses in a litter was calculated after 24 (for doses<150 µg/Kg) or 48 h (200 µg/Kg). LPS (20 to 120 µg/kg) did not induce any fetal loss (data not shown). LPS (150 µg/kg) resulted in fetal death of ∼7%, while LPS (200 µg/kg) caused >20% fetal death (Table 1). LPS (150 µg/kg) was subsequently used for acute functional and expression studies.

**Table 1:** Rate of fetal death/reabsorption after acute LPS exposure.

LPSInjection Day	Groups(LPS µg/Kg)		Exposureh)	EuthanasiaDay	N(dams)	% of fetal death/reabsorption
E15.5	Vehicle150		44	E15.5	65	0 (0/56)0 (0/39)
E14.5	Vehicle150		2424	E15.5	510	0 (0/42)**7.9** (5/63)
E17.5	Vehicle150		44	E17.5	55	0 (0/40)0 (0/36)
E16.5	Vehicle150		2424	E17.5	44	0 (0/32)0 (0/32)
E14.5	Vehicle		48	E16.5	3	3,3 (1/30)
	200	48			2	**52.2** (10/19)

For the chronic experiments, pregnant mice received LPS (5 or 35µg/Kg, i.p.) every 24 h from E11.5 to E15.5. Maternal dissection was performed and fetal survival rates were evaluated 4 h after the final LPS treatment. Chronic LPS (35µg/Kg) caused ∼90% fetal death/reabsorption, while 5µg/Kg produced ∼15% fetal loss (Table 2). As a result, 5µg/Kg LPS was selected for chronic functional and expression studies.

**Table 2:** Rate of of fetal death/reabsorption after multiple LPS exposure.

LPSexposure	Gestationalage	Groups(LPS µg/Kg)	Total exposuretime (hs)	N(dams)	% of fetal death/reabsorption
		Vehicle		7	3,7 (1/55)
Multiple	E15.5	5	124*	8	**15.8** (9/57)
		35		3	**88.5** (23/26)

* Dams received daily Veh/LPS treatments from E11.5 until E15.5 and were euthanized 4 hs after last treatment on E15.5.

### 2.3. Animal Experimentation

After determining LPS treatments that cause minimal fetal death, acute and chronic LPS treatments were conducted. The embryonic time points were selected as we have previously demonstrated that in the mouse placenta, P-gp expression peaks at E12.5 and progressively declines towards term [8], [13], [17]. Furthermore, the mouse chorioallantoic placenta is established by E12.5 [24], which marks the onset of fetal dependence upon placental uptake of nutrients from the maternal blood. Therefore, from E11.5, LPS-induced changes in P-gp expression/activity would be more likely to impact fetal drug exposure. The 4 & 24 h time-points were selected as previous studies have shown that very high doses of LPS inhibit *Abcb1a/b* expression and activity at this time point in rats [6], [7].

Acute experiments were performed by injecting LPS (150 µg/Kg) or vehicle (saline; i.p.), 4 h or 24 h prior to maternal euthanasia on E15.5. Whereas, chronic experiments were performed by injecting LPS (5 µg/Kg) or vehicle daily from E11.5-E15.5 (i.p.). Maternal euthanasia was performed 4 h after the final LPS challenge. Euthanasia was performed by lethal dose of isoflurane inhalation followed by cervical dislocation; fetuses were euthanized by decapitation.

The entire fetal unit including the placenta were removed from the gravid uterine horns and dissected immediately as previously described [17]. Briefly, intact placental discs were mechanically detached from the fetal units (comprising of the fetus, amniotic fluid and intact fetal membranes) and were weighed and processed separately. Placental and fetal-unit wet weights were recorded and each placenta was immediately placed in RNALater (QIAGEN, Valencia, CA) for use in real-time PCR (qPCR) measurements. Maternal plasma was collected by cardiac puncture in heparinized tubes, kept on ice and plasma collected following centrifugation (1077 g, 15 min). Maternal plasma was frozen at −80°C until further analysis. Additionally, in the chronic LPS and vehicle treated groups, maternal splenic wet weight was recorded to assess whether chronic LPS exposure would result in maternal splenomegaly (an indicator of chronic maternal inflammation).

### 2.4. ELISA

In order to control for the presence of maternal inflammatory response after LPS treatments, measurements of the pro-inflammatory cytokine interleukin 6 (IL-6) in maternal plasma were performed by ELISA using a commercially available kit (R&D systems, Minneapolis, MN, USA) in accordance with the manufacturer’s instructions (minimum detectable dose = 1.6 pg/mL; intra-assay variation = 4.7%).

### 2.5. In vivo Distribution of [^3^H]digoxin

In another set of animals (n = 49), functional studies testing P-gp activity after LPS challenge were performed by injecting a mixture of cold digoxin (50 µg/kg; Sigma) and [^3^H]digoxin (1µCi/pregnant dam; 29.8 Ci/mmol - Perkin-Elmer, Boston, Massachusetts, USA) directly into maternal tail vein. Digoxin has been extensively used to assess P-gp function as previously described by our group and others [17], [23], [25], [26]. Briefly, the digoxin mixture was injected 1 h prior to euthanasia with a lethal dose of isoflurane inhalation, followed by heart puncture. Maternal blood was collected via cardiac puncture and plasma was separated for further analysis. After dissection, placentas and fetal-units (comprised of fetus and all fetal membranes and amniotic fluid) were collected and weighed. To determine net transplacental transfer, fetal-units were collected in one of two ways: a) Acute LPS: four placentas/litters were arbitrarily collected and averaged per dam to provide a litter mean that was used for statistical analysis and, b) Chronic LPS: fetal-units in each litter were collected and further analyzed. Maternal hearts were collected, weighed and processed (described below), in order to evaluate whether LPS exposure targets P-gp activity in a tissue-dependent manner.

All samples were homogenized in PBS. Then fetal-units and maternal myocardial aliquots (200 µl) were solubilized in SOLVABLE (1 ml; PerkinElmer) followed by the addition of hydrogen peroxide (30%; 100 µl - Sigma) to decolorize samples and optimize counting efficiency. Scintillation fluid (10 ml) was then added (Ultima-Gold, PerkinElmer) and radioactivity (disintegrations per minute; DPM) was quantified on a Tri-Carb Beta-Counter (PerkinElmer). P-gp-mediated transplacental transfer was calculated as a ratio of radioactivity present in the fetal-unit (DPM) relative to maternal plasma (DPM) standardized per gram of placenta [23]. This method provides a direct measure of P-gp substrate accumulation per gram of placental tissue.

### 2.6. RNA Extraction, cDNA Preparation, and qPCR Analysis

For the acute experiments, one placenta/litter was arbitrarily collected and immediately stored in RNALater (QIAGEN). For the chronic experiments, three placentas/litter were collected to evaluate if P-gp expression is placental size-dependent: the lightest wet-weight placenta (small), a placenta closest to the mean wet weight in a litter (middle) and the placenta with the heaviest wet-weight (large). Total RNA was extracted using TRIZOL reagent (Invitrogen) according to the manufacturer’s instructions. Samples were digested with a ribonuclease-free deoxyribonuclease (Ambion-Austin, TX). RNA purity and concentration were assessed by the A260/A280 ratio using spectrophotometric analysis and RNA integrity was verified using gel electrophoresis. Total RNA was converted to cDNA using Multiscribe Reverse Transcriptase (50 U/µl), deoxynucleotide triphosphate mix and random (hexameric) primers (Applied Biosystems,Foster City,CA). For the acute LPS exposure qPCR experiments, 50 ng/µl of total RNA was converted to cDNA, whereas for the chronic LPS exposure qPCR experiments, 300 µg/nl of total RNA was used. Samples were incubated at 25°C for 10 min, 37°C for 120 min, and 85°C for 5 min using the C1000 Thermal Cycler (Bio-Rad, Hercules, CA).

qPCR was performed using the C1000 Thermal Cycler and quantified using the CFX96 Real-Time System (Bio-Rad). Samples were run in triplicate and prepared using TaqMan Universal PCR Master Mix (Applied Biosystems, Hammonton, NJ), and a non-template negative control was included in all runs using water instead of cDNA.

We ran primer-probes set for the following genes: A) the reference genes, glyceraldehyde 3-phosphate dehydrogenase - *Gapdh* (lot no. 4352932E), TATA box binding protein - *Tbp* (Mm00446971_m1) and hypoxanthine-guanine phosphoribosyltransferase - *Hprt* (Mm01545399_m1); B) the multidrug resistance encoding genes, *Abcb1a* (Mm00440736_m1), *Abcb1b* (Mm00440761_m1), *Abcg2* (Mm00496364_m1); C) the pro- inflammatory cytokines, interleukin *(Il)-6* (Mm00446190_m1) and tumor necrosis factor-alpha (*Tnf-α)* (Mn00443260_g1); D) the anti-inflammatory cytokine *Il-10* (Mm00439614_m1) E) the *toll-like receptor-4* (*Tlr-4*; Mm00445273_m1) (Applied Biosystems), and cDNA template (50 ng) using ratios according to manufactures instructions (initial denaturation at 95°C for 20s), followed by 40 cycles of 95°C for 1s and 60°C for 20s) in a final reaction volume of 10 µl. Resulting data were analyzed using CFX Manager Software (Bio-Rad).

Relative gene expression was calculated by the [ΔΔc(t)] method [27], normalized to reference gene expression (*Tbp, Gapdh* and or *Hprt*) [28]. We have previously used *Tbp* and *Gapdh* as reference genes in the mouse placenta [17], [22], [29]. Therefore, we tested whether expression of these genes was stable after acute or chronic LPS treatment. Additionally, we tested whether *Hprt* mRNA levels were stable in placentas of different size, exposed to chronic sub-lethal LPS. *Tbp*, *Gapdh* and *Hprt* mRNA expression was similar among groups, therefore validating their use as reference genes in the present study.

### 2.8. Statistical Analysis

Values for all data are expressed as mean±SEM and were analyzed using Prism (GraphPad Software Inc., San Diego, CA). After confirming normal distribution, differences between vehicle and LPS treated groups were assessed by one-way ANOVA followed by the Tukey’s post-hoc test; or otherwise by the Kruskal-Wallis analysis of variance followed by Dunn’s test for non-parametric comparisons. Pearson’s correlation test was used to assess correlation between placental weight and fetal [^3^H]digoxin accumulation. Repeated measures (mixed model) two-way ANOVA followed by Bonferonni’s test was used to compare the effect of chronic LPS-treatments on [3H]digoxin accumulation and mRNA expression in different sized-placenta. For this statistical model, we designated placental size differences from each individual dam as the repeated measures factor (i.e. within litter variations were calculated by matching smaller, mid-range and larger placentas values from each dam). Between litter variations were calculated by comparing matched placental values exposed to sub-lethal chronic LPS-treatments or vehicle. Statistical significance was considered if p<0.05.

## Results

### 3.1. Acute sub-lethal LPS: Maternal Inflammatory Response

**Figure 1:**
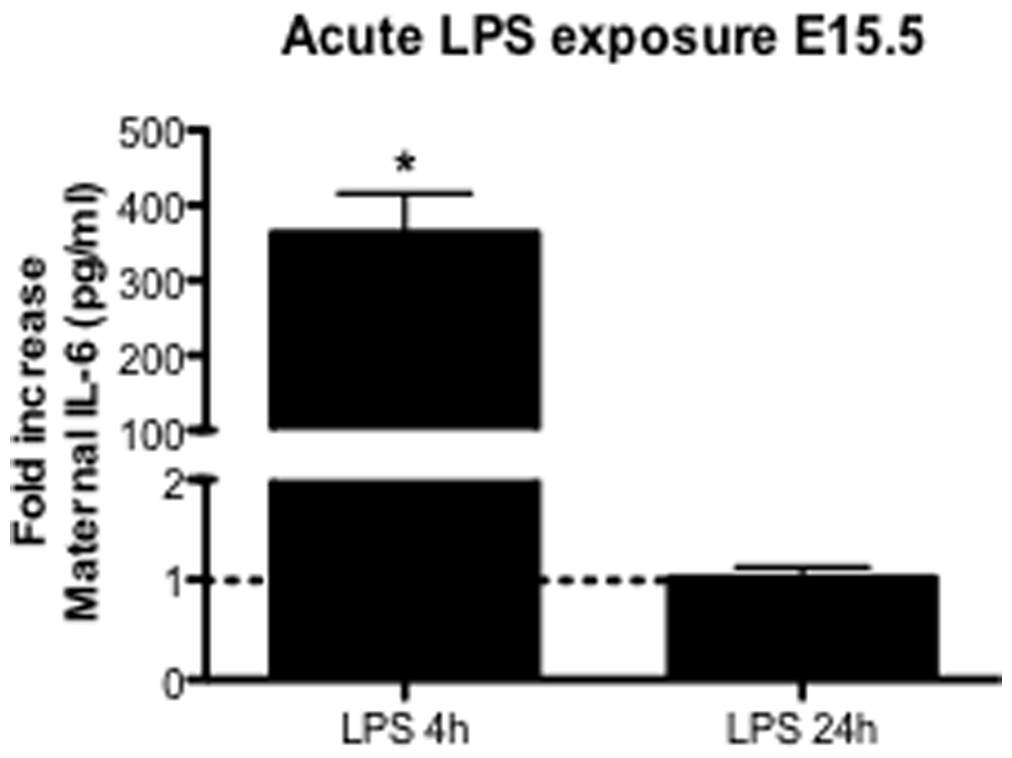
Maternal IL-6 plasma levels in mice exposed to acute LPS treatment. Vehicle and acute LPS (150 µg/Kg) treatments were performed and maternal plasma was extracted: 4 h (n = 6/gp) or 24 h (n = 5/gp) after single LPS exposure. Values are means±SEM. ***p<0.0001 (one-way ANOVA followed by the Tukey’s post-hoc test).

IL-6 levels were measured in the maternal plasma 4 or 24 h after single LPS treatment on E15.5. IL-6 measurements were conducted since it is a sensitive marker of systemic inflammation and preterm delivery (PTD), moreover, IL-6 is highly augmented after LPS [30], [31]. IL-6 levels were ∼ 360 times higher than control 4 h after acute LPS treatment (150 µg/kg; *p*<0.001) but returned to baseline after 24 h (Fig. 1). These results demonstrate that our selected dose elicits a maternal cytokine inflammatory response without significant fetal loss.

### 3.2. Decreased Placental P-glycoprotein Activity after Acute sub-lethal LPS Exposure

**Figure 2:**
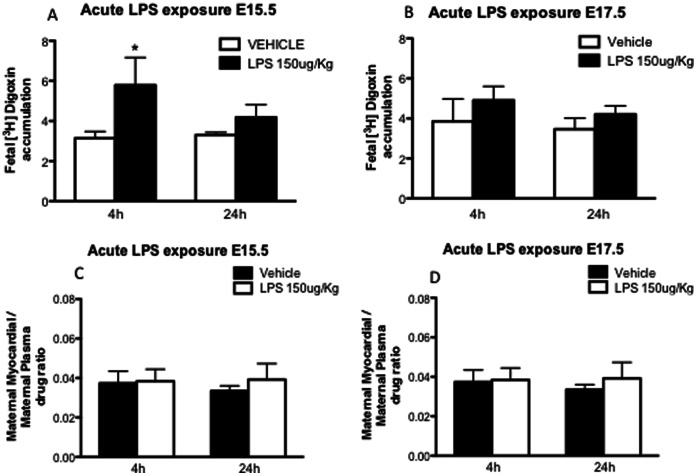
Placental and maternal myocardial P-gp activity after acute sub-lethal LPS exposure: Fetal Units [^3^H]digoxin accumulation (4 fetal units/dam were randomly harvested and assayed) on E15.5 (A) (n = 5 dams/gp) and E17.5 (B) (4 h n = 5 dams/gp; 24 h n = 4 dams/gp); 4 or 24 h after acute LPS treatment. Maternal myocardial [^3^H]digoxin accumulation on E15.5 (C) and E17.5 (D) 4 or 24 h after acute LPS treatment. Values are means±SEM. *p<0.05 (one-way ANOVA followed by the Tukey’s post-hoc test on E15.5 and Kruskal-Wallis analysis of variance followed by Dunn’s test on E17.5).

**Figure 3:**
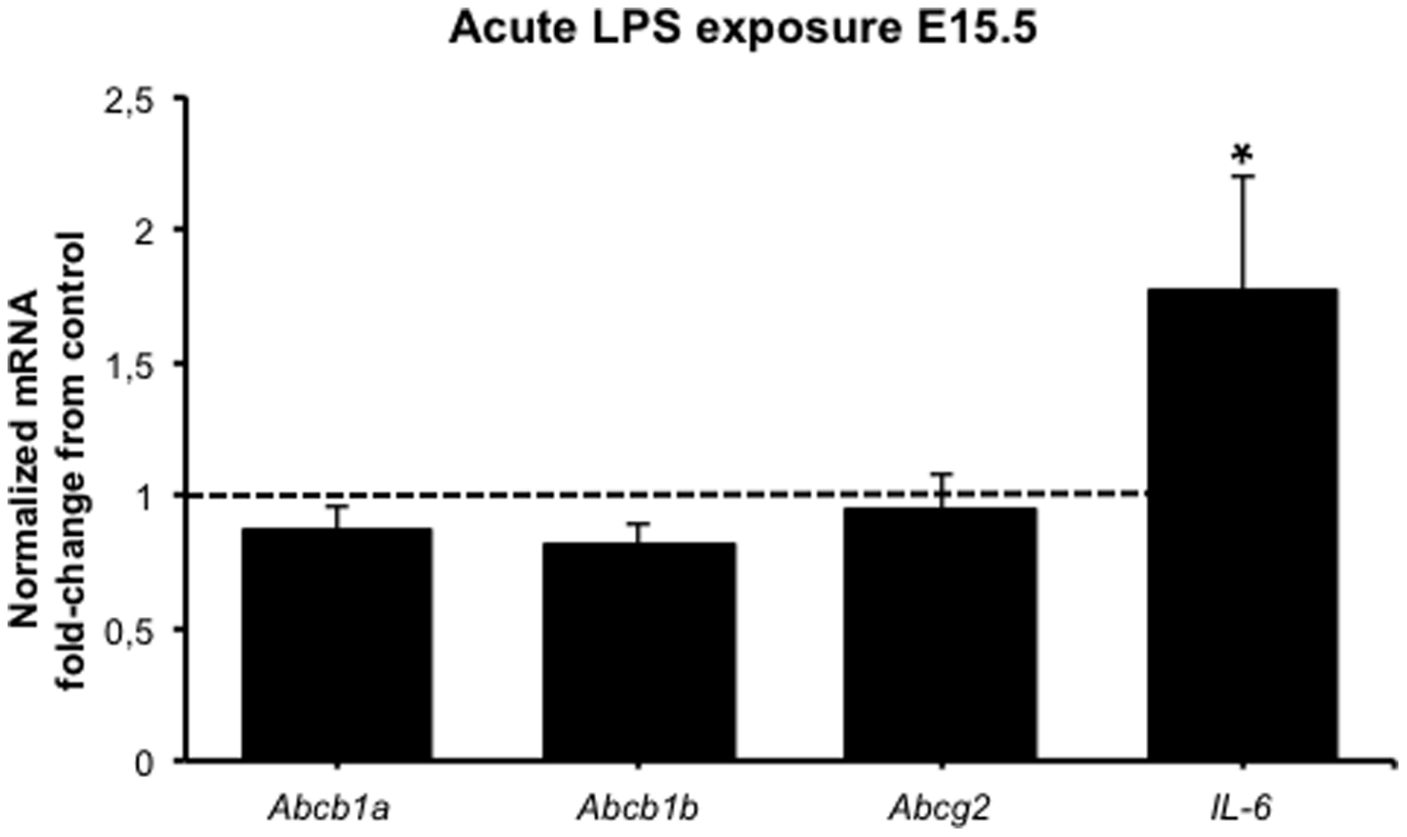
Placental mRNA expression of the multidrug resistance genes (Abcb1a, Abcb1b, and Abcg2) and the pro-inflammatory cytokine Il-6 after acute sub-lethal LPS exposure: one placenta per group was randomly harvested 4 h after LPS insult and processed for qPCR analyses (n = 6 dams/gp). Values are fold increase of the means±SEM. *p<0.05 (one-way ANOVA followed by the Tukey’s post-hoc test). Relative gene expression normalized to Tbp Gapdh.

**Figure 4:**
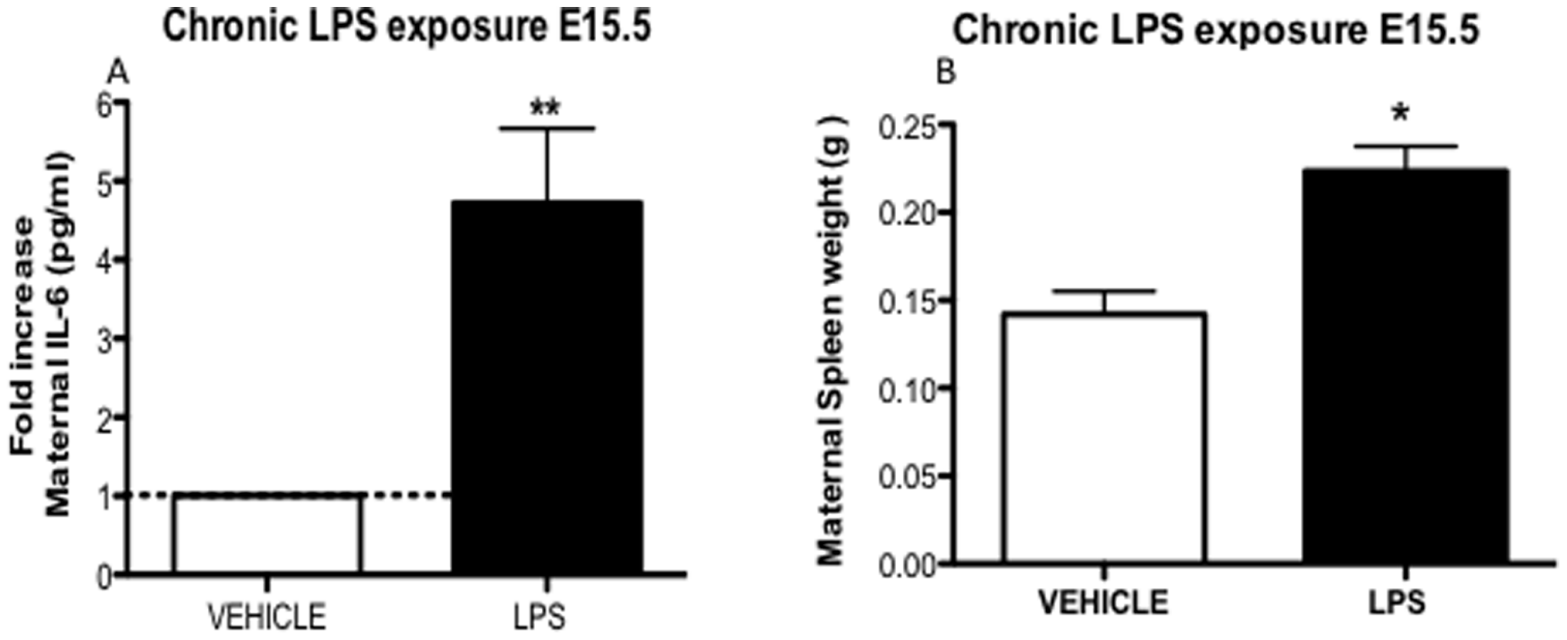
Maternal IL-6 plasma levels and spleen weight in mice exposed to chronic LPS treatments. Chronic LPS (5 µg/Kg) treatment was performed daily from E11.5 until E15.5. (A) Maternal plasma was extracted 4 h after last LPS treatment on E15.5 (Vehicle n = 9; LPS n = 10). (B) Maternal splenic weight from animals exposed to chronic LPS treatment (Vehicle n = 9; LPS n = 10). Values are means±SEM. *P<0.05, **p<0.001 (one-way ANOVA followed by the Tukey’s post-hoc test).

P-gp activity was assessed by measurement of [^3^H]digoxin accumulation in fetal-units on E15.5 (Fig. 2A) and E17.5 (Fig. 2B), 4 or 24 h after the inflammatory insult. On E15.5, [^3^H]digoxin accumulation was increased 4 h after acute LPS insult (*p*<0.05), whereas no differences were found 24 h after LPS exposure. No differences in fetal-unit [^3^H]digoxin accumulation were found on E17.5. Maternal myocardial P-gp activity was not different at any time point analyzed (Figs 2C/D). *Abcb1a* and *Abcb1b* (genes encoding P-gp expression in the mouse) and *Abcg2* (encoding BCRP) mRNA levels were unaltered 4 h after acute sub-lethal LPS exposure, whereas *Il-6* transcripts were ∼ 2-fold increased (*p*<0.05) compared to controls (Fig. 3).

### 3.3. Chronic Sub-lethal LPS: Maternal Inflammatory Response

Chronic LPS treatment (5 µg/Kg) produced a 5-fold increase (*p*<0.01) in maternal plasma IL-6 4 h after the final chronic LPS treatment on E15.5 (Fig. 4A). Furthermore, we found marked maternal splenomegaly (*p*<0.01) when compared to controls (Fig. 4B), showing the presence of recurrent maternal immune system activation and systemic inflammation during treatment.

### 3.4. Tissue Specific Changes in P-glycoprotein Activity after Chronic LPS Exposure

**Figure 5:**
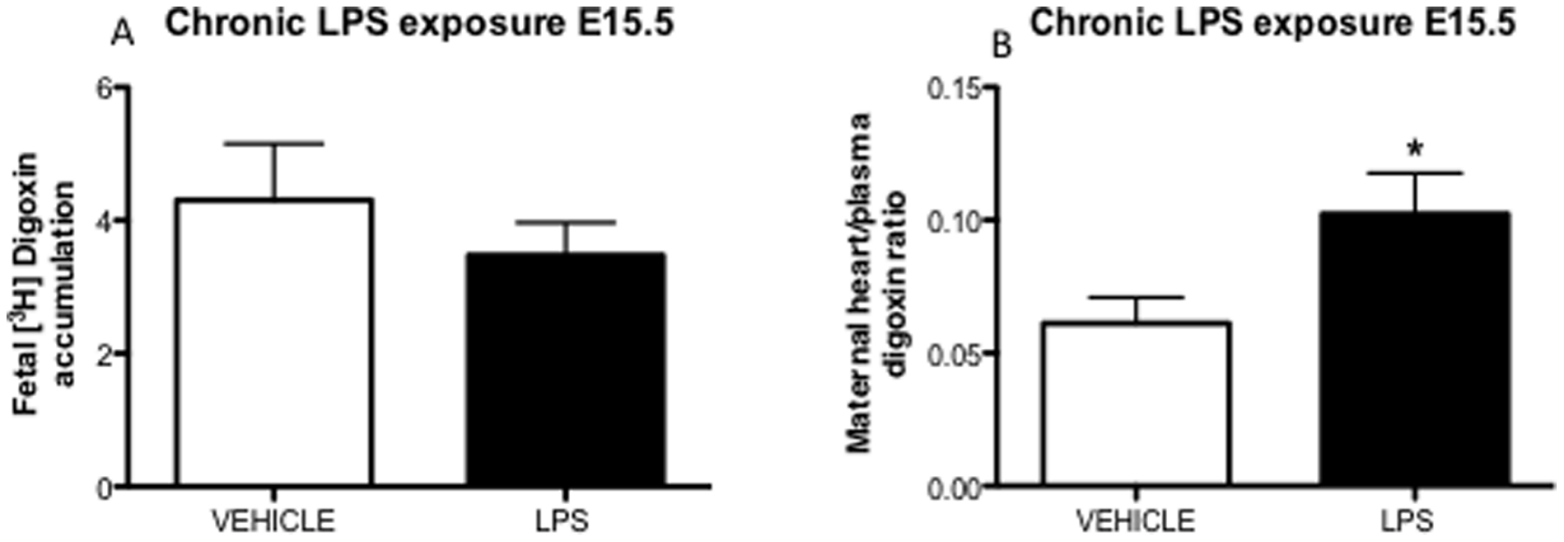
Placental and maternal myocardial P-gp activity after chronic sub-lethal LPS exposure. (A) fetal units [^3^H]digoxin accumulation (all fetuses/dam) and (B) maternal myocardial [^3^H]digoxin accumulation, 4 h after last LPS chronic treatment (daily from E11.5 until E15.5) on E15.5 (vehicle n = 5; chronic LPS n = 6). Values are means±SEM. *P = 0.05 (one-way ANOVA followed by the Tukey’s post-hoc test).

Since there was ∼15% fetal death/reabsorption after chronic LPS treatment, all the fetal-units per dam were collected and analyzed for [^3^H]digoxin accumulation. There were no differences in the overall fetal drug accumulation after chronic LPS treatment compared to controls (Fig. 5A). Conversely, maternal myocardial digoxin accumulation (Fig. 5B) was increased (*p*<0.05), demonstrating that sub-lethal LPS exposure decreases P-gp activity in a tissue-specific manner.

### 3.5. Placental Size and P-gp Transport Efficiency

**Figure 6:**
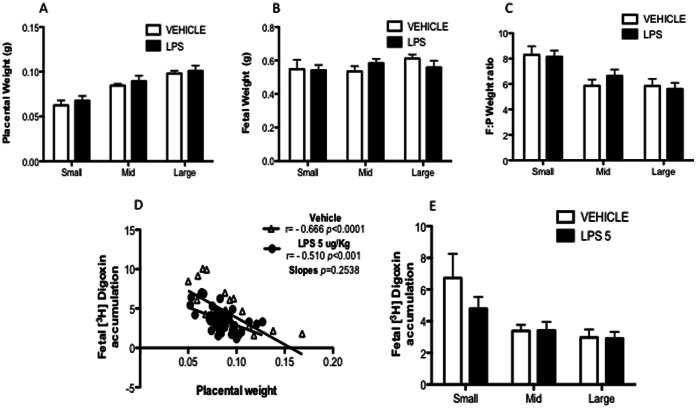
Placental size and P-gp transport efficiency. Small, mid-range and larger placentas from each litter were grouped and averaged for placental (A) and fetal (B) weight and fetal to placental (F:P) weight ratio (C). (D) shows the relationship between individual placental weights and [^3^H]digoxin fetal accumulation at E15.5, for vehicle (r = −0.6657, n = 31 fetuses from 5 litters, *p*<0.0001) and chronic LPS (r = −0.5099, n = 39 fetuses from 6 litters, *p*<0.001) groups. (E) [^3^H]digoxin fetal accumulation from small, mid-range and larger placentas at E15.5 (vehicle n = 5; LPS n = 6). (A,B,C and E, repeated measures two-way ANOVA followed by Bonferonni’s test, *p*<0.05; Pearson’s correlation test).

In order to evaluate whether P-gp transport efficiency varies according to placental size and F:P weight ratio, all fetal-units per dam were assayed for [^3^H]digoxin accumulation on E15.5. There were no differences in placental and fetal weight or F:P weight ratio after chronic LPS exposure (between-litter variability) (Fig. 6A–C). As hypothesized, the repeated measures two-way ANOVA revealed a significant within-litter variability in placental weight (p<0.0001) and F:P weight ratio (p<0.0001).

The relationship between placental size and placental P-gp activity was then analyzed (Fig. 6D). There was an inverse relationship between placental size and P-gp activity. Larger placentas were more efficient in effluxing [^3^H]digoxin than smaller placentas in both vehicle (r = −0.666; *p*<0.0001) and chronic LPS treated (r = −0.510; *p*<0.001) groups. The correlation slopes did not differ (*p* = 0.253). There were no between-litter effects when comparing [^3^H]digoxin accumulation in same sized-placentas exposed to chronic sub-lethal LPS treatments. Conversely, within-litter variability was detected for placental size (p<0.001), which accounted for ∼ 35% of the total variance, showing that P-gp activity differs significantly according to placental size.

### 3.6. Placental Size and LPS-specific Effects on Multidrug Resistance and Cytokine Expression

**Figure 7:**
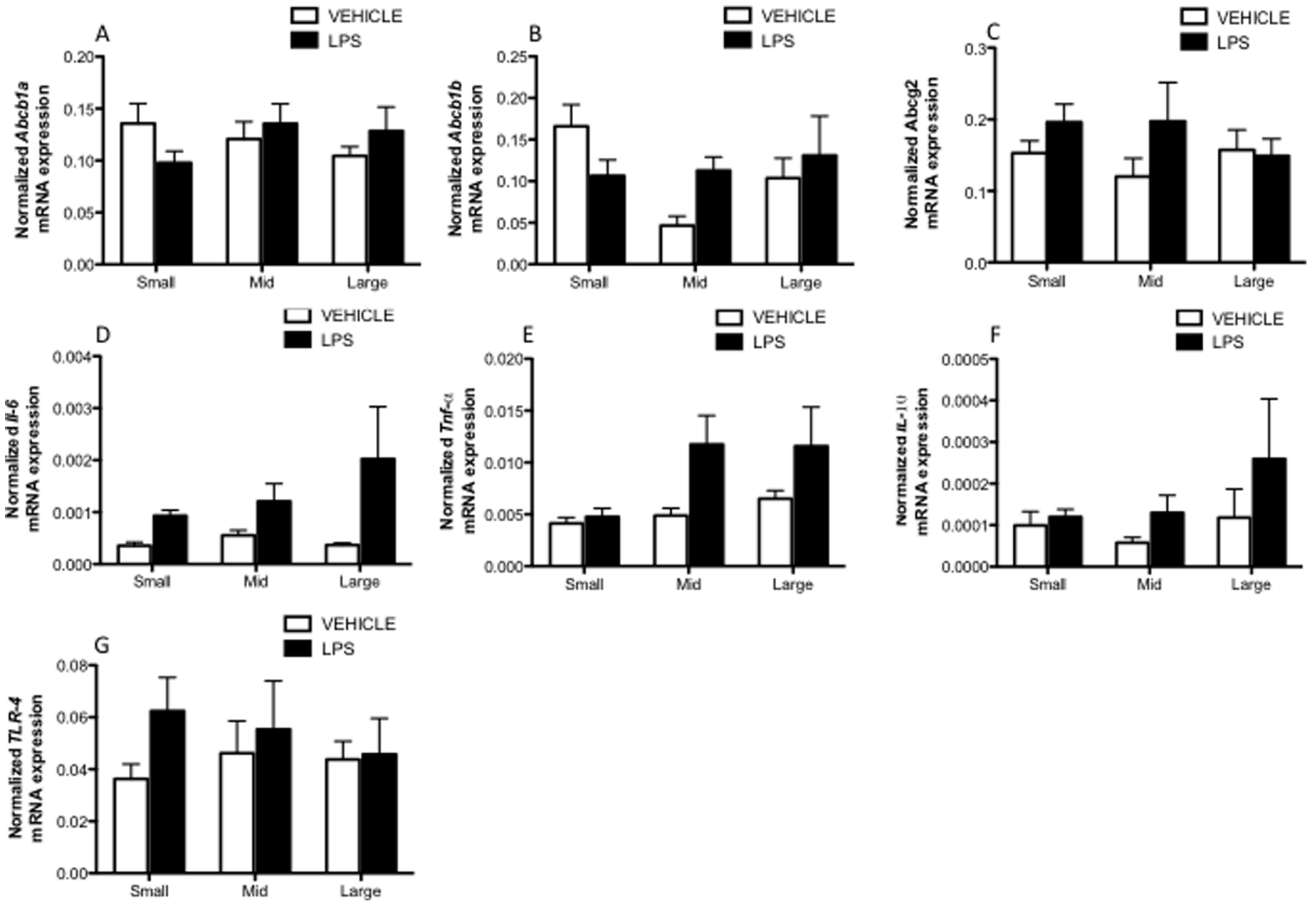
Placental mRNA expression of the multidrug resistance genes (Abcb1a, Abcb1b, and Abcg2), Il-6, Tnf-α, Il-10 and Tlr-4 after chronic LPS exposure. The small, mid-range and larger placentas from each litter were grouped and assayed for mRNA expression. (Veh, n = 7 dams; LPS n = 7). (Repeated measures two-way ANOVA followed by Bonferonni’s test, *p*<0.05). Relative gene expression normalized to *Tbp, Gapdh* and *Hprt*.

To determine if chronic LPS exposure elicits changes in placental expression of selected cytokines (*Il-6, Tnf-*α and *Il-10*), *Tlr-4* and multidrug resistance-encoding genes (*Abcb1a*, *Abcb1b* and *Abcg2)*, qPCR was performed in the smaller, mid-range and larger placentas (Fig. 7A–G). Repeated measures two-way ANOVA revealed that LPS effectively induced placental *Il-6* and *Tnf-α* mRNA expression (p<0.05; between-litter effect). However, LPS-induced *Tnf-α* expression was placental size-dependent (p<0.05; within-litter effect), since smaller placentas from endotoxemic mothers did not exhibit a *Tnf-α* response. There were no placental size-related changes in multidrug resistance-encoding genes, *Abcb1a, Abcb1b* and *Abcg2*, after chronic LPS exposure.

## Discussion

In order to examine whether placental drug transport is altered in cases where PTD does not occur following maternal infection, we conducted a series of experiments in a well-characterized murine model of sub-lethal infected pregnancies. We have shown that acute sub-lethal (fetal) infection increases fetal drug accumulation in the mouse in a time- and gestational age-dependent manner. Furthermore, we discovered that P-gp activity and placental responsiveness to inflammatory insult vary according to placental size.

Among the 11 members of the mammalian TLR family that have been identified to date, TLR2 and TLR4 are the major receptors involved in recognition of bacterial cell wall components [32]. In third trimester human placenta, higher levels of TLR2 were found in endothelial cells and macrophages (with low levels of TLR2 in the syncytiotrophoblast). In contrast, high levels of TLR4 were present in the syncytiotrophoblast [32], [33], [34]. TLR-4 is highly expressed in preterm human placentas from complicated chorioamnionitis [33] and has been extensively associated with PTD [3]. Importantly, P-gp and BCRP are also highly expressed in the syncytiotrophoblast [13]–[16]. Thus, in the present study we examined TLR-4 activation in a LPS regimen to mimic sub-lethal acute and chronic infections during pregnancy.

Acute treatment with 150 µg/Kg LPS promoted increased fetal loss when given earlier in gestation (E14.5 vs. E17.5), indicating that fetuses are more susceptible to acute infection during early stages of gestation. As hypothesized, acute LPS treatment resulted in increased systemic maternal IL-6 4 h following exposure, but returned to baseline 24 h later. This corroborates previous findings showing time-dependent LPS-induced IL-6 secretion [30] and demonstrating a marked systemic inflammatory response in the mother. In parallel, placental P-gp activity was decreased 4 h following acute LPS but returned to baseline after 24 h, suggesting that increases in systemic pro-inflammatory cytokines, such as IL-6, regulate placental P-gp activity. A placental inflammatory response was also indicated by the increased placental *Il-6* mRNA expression. The fact that increased fetal drug accumulation following acute LPS insult is not followed by changes in placental multidrug resistance gene expression indicates that there can be changes in placental P-gp activity independent of changes in *Abcb1a/b* gene expression. A disconnect between *Abcb1a/b* gene expression and P-gp activity has been previously demonstrated in the murine placenta [17]. Interestingly, placental P-gp activity was altered by acute LPS on E15.5 but not E17.5, demonstrating that changes in P-gp activity elicited by acute sub-lethal inflammatory insult are more likely to occur in early stages of pregnancy. In addition, it is possible that changes in placental P-gp expression across pregnancy may result in differences in P-gp sensitivity to inflammatory stimuli. In fact, placental P-gp expression peaks at E12.5 and progressively declines towards term [8], [13], [17]. P-gp sensitivity to inflammatory insult was also tissue-specific since acute LPS exposure did not change maternal myocardial P-gp activity, demonstrating that inflammation targets P-gp activity in a tissue-dependent manner.

With respect to chronic sub-lethal LPS exposure, lower doses (5 µg/Kg; E11.5–15.5) promoted two-fold higher fetal loss compared to a single LPS treatment (150 µg/Kg; E14.5), demonstrating that chronic infections have a greater negative impact on pregnancy than acute infections. One possible reason for increased fetal loss in the lower dose regimen may be due to the fact that chronic LPS treatment was initiated at an earlier stage of pregnancy than in the acute experiments. Chronic LPS treatment also promoted increases in systemic maternal levels of IL-6. Moreover, profound splenomegaly was found in the chronic LPS treated dams. Splenomegaly is an important indicator of recurrent innate and adaptive immune response activation. It commonly develops in systemic infections caused by chronic gram negative bacteria-mediated infections in rodents and humans [35], [36], [37].

The [^3^H]digoxin accumulation assay revealed there was no difference in the overall placental P-gp activity after chronic LPS exposure. Conversely, maternal myocardial [^3^H]digoxin accumulation was significantly increased, again demonstrating tissue-specific changes in P-gp activity during chronic infection. Importantly, tissue-specific changes in P-gp expression have been previously reported during intermittent hypoxia on myocardium and liver [38], indicating that P-gp response to infections can differ throughout the body.

Our results demonstrate that the smaller placentas in a litter are less efficient at effluxing P-gp substrates than larger placentas. A similar pattern was found in the chronically infected fetuses, although the correlation was not as strong. This demonstrates that chronic infection does not change the relationship between placental size and placental drug transport efficiency. The finding that placental P-gp activity is decreased in the smaller placentas contrasts that described for the placental nutrient transporters. In this case, smaller placentas are more efficient in transporting system A substrates per gram of placenta when compared to their larger counterparts [19], [21], [22]. Such adaptations are very important to promote proper fetal growth in the smaller placenta. However, since we found that smaller placentas are less efficient at extruding P-gp substrate per gram of placenta, it is clear that placental drug transporter efficiency does not follow the same trend as that previously described for nutrient influx transporters. Functionally, it may result in increased transfer of xenobiotics from the mother to fetuses with a smaller placenta.

Despite the fact that the smaller placentas within a litter transport nutrients more efficiently, smaller placentas display reduced numbers of fetal capillaries in the labyrinth zone [21]. Consequently, they receive less blood flow than their larger counterparts. Interestingly, intestinal ischemia/reperfusion decreased P-gp-mediated ileal excretion of rhodamine 123 (a P-gp substrate) [39]. Further, cerebral ischemia/reperfusion increases P-gp substrate accumulation in the brain of rats [40]. Together, these findings would suggest that decreases in local blood flow could decrease P-gp activity. Reduced placental P-gp activity in small placentas, as occurs in IUGR/pre-eclamptic pregnancies and in pathologies resulting from uteroplacental vascular diseases, may increase fetal exposure to harmful substances and xenobiotics present in the maternal circulation. This impairment in P-gp activity in response to decreased or compromised circulation could have a wide variety of implications for neonatal health.

Differences in placental *Abcb1* (P-gp encoding gene in the human) and *Abcg2* (BCRP) mRNA expression-associated with infection and inflammation have been previously reported in human placenta. Treatment with the pro-inflammatory cytokines TNF-α and IL-1β decreased *Abcb1* and *Abcg2* mRNA expression in human primary trophoblast cultures [41]. Accordingly, LPS decreased *Abcb1a/b* and *Abcg2* mRNA in rats during late pregnancy [6], [7], whereas, *Abcb1* and *Abcg2* mRNA were upregulated in cases of PTD with confirmed inflammation [10]. These divergent findings suggest that there are species-specific factors determining P-gp response to infection/inflammation, or alternatively that P-gp responds differently according to the type of the infective/inflammatory stimulus. In the present study, we report that there were no changes in *Abcb1a* and *Abcb1b* mRNA expression after acute or chronic sub-lethal LPS exposure. We were careful to titrate a dose of LPS that did not induce PTD after 24 hours. It is possible that if we had used a higher dose of LPS, as was previously used in the rat [7], there may have been significant changes in *Abcb1a*, *Abcb1b* and *Abcg2 mRNA.* It is also possible that if we had modeled intrauterine infection (through injection of LPS directly into the fetal compartment) or administered TNF-α, IL-1β or IL-6 directly to the mother, we would also have seen altered placental multidrug resistance.

It is important to note that inflammatory responses are mediated by a complex interplay of cytokines, chemokines, prostaglandins, leukotrienes, and complement [42]. Indeed, many obstetric diseases and neonatal outcomes/disorders may be caused by different patterns of cytokines, chemokines, soluble receptors, or growth factors [42]. In this regard, it is possible that additional pathogen-related components, through simultaneous activation of different pattern recognition receptors (PPRs) and or different intracellular pathways, may be required to induce changes in multidrug resistance expression and activity in chronic sub-lethal infections. Further studies are required to investigate these possibilities.

Placental responsiveness to chronic inflammatory insult was also size-dependent. After LPS challenge, levels of *Tnf-α* mRNA were increased only in the mid-range and larger placentas but not in the smaller placentas, suggesting that a larger placenta is more efficient in mounting a selective pro-inflammatory response. Of note, *Tnf-α* is potently induced by LPS in human primary trophoblast cell cultures and villous explants [43], [44]. Furthermore, at the protein level TNF-α has been closely associated with intrauterine infection and pre-term labor [3]. This finding led us to postulate that *Tlr-4* expression, which is a major receptor of the innate immune system and is highly responsive to gram negative bacteria [45], may differ according to placental size. Which could explain differences in pro-inflammatory cytokine expression. *Tlr-4* transcript was unchanged in different sized-placentas after chronic LPS exposure. Anti-inflammatory cytokines also play a major role in reducing the severity of TLR-mediated inflammatory responses to different inflammatory agents in the placenta [46]. Importantly, *IL-10* is highly responsive to LPS in the human placenta [44], [47]; therefore placental size-dependent changes in *IL-10* expression could underlie differences in placental-size dependent responsiveness to LPS. No differences in *Il-10* mRNA were found after chronic LPS exposure.

In summary, we have provided the first evidence that acute sub-lethal infection decreases placental P-gp activity in a time- and gestational age-dependent manner, and that chronic infection does not change overall placental P-gp expression and activity. We have also demonstrated that placental P-gp activity and immunological responses differ according to placental size, emphasizing the need for more studies evaluating whether fetuses with smaller placentas are at increaseed risk of exposure to xenobiotics and harmful substances present in the maternal circulation. This may have implications in pathological conditions of IUGR or PE which have growth restricted placentas. Clearly, focused studies must now be undertaken in the human placenta to determine the effects of systemic and localized intrauterine infection on multidrug resistance in the placenta and fetus.
